# Behavioral economics-based incentives supported by mobile technology on HIV knowledge and testing frequency among Latino/a men who have sex with men and transgender women: Protocol for a randomized pilot study to test intervention feasibility and acceptability

**DOI:** 10.1186/s13063-018-2867-1

**Published:** 2018-10-05

**Authors:** Sebastian Linnemayr, Sarah MacCarthy, Alice Kim, Rebecca Giguere, Alex Carballo-Dieguez, Joanna L. Barreras

**Affiliations:** 1 RAND Corporation, Economics, Sociology, and Statistics, 1776 Main Street, Santa Monica, CA USA; 2 RAND Corporation, Behavioral and Policy Sciences, 1776 Main Street, Santa Monica, CA USA; 3 HIV Center for Clinical and Behavioral Studies, Division of Gender, Health and Sexuality, New York State Psychiatric Institute and Columbia University, 1051 Riverside Drive, Unit 15, New York, NY USA; 4 Bienestar Human Services, Inc., 5326 East Beverly Blvd, Los Angeles, CA 90022 USA

**Keywords:** Feasibility and acceptability, HIV testing, Mobile technology, Behavioral economics, Intervention, Incentives, Latino, Men who have sex with men, Transgender women

## Abstract

**Background:**

Mobile Technology and Incentives (MOTIVES) is a randomized pilot study of a mobile technology-based and behavioral economics-supported HIV prevention intervention. Behavioral economics (BE) uses financial incentives in a way that departs from the traditional focus on large monetary payments. Instead, BE suggests that relatively small “nudges” can effectively initiate and sustain behavior change. This pilot study examines the feasibility and acceptability of an HIV prevention intervention that uses text messages in combination with BE incentives to improve retention of HIV prevention information and increase frequency of HIV testing among Latino/a men who have sex with men (MSM) and transgender women (TGW). The pilot will also estimate mission-critical design parameters with point and confidence interval estimates of the intervention to inform a future, fully powered effectiveness study.

**Methods:**

The project will be conducted in collaboration with Bienestar Human Services, Inc. (*Bienestar*), a non-profit community-based service organization. The intervention is being tested in a small, randomized controlled trial to pilot the intervention’s feasibility and acceptability among 200 Latino/a MSM and TGW from *Bienestar*’s HIV testing sites. Information on feasibility will include recruitment, refusal, and retention rates as well as message sending success rates; acceptability will include perceived appropriateness based on responses to the intervention. Participants will be randomized into either the “information only” control group (e.g. receiving text messages with HIV prevention information) or the “information plus” intervention group (e.g. additionally receiving quiz questions that provide the possibility of winning prizes). Participants will be followed for 12 months from enrollment. In addition to using data abstracted from *Bienestar*’s routine data collection mechanisms, we will also collect survey data (blinded outcome assessment) from participants at 0, 6, and 12 months to provide an initial assessment of whether incentives affect their level of HIV knowledge and testing frequency.

**Discussion:**

If shown to be acceptable, feasible, and resource-efficient, MOTIVES will provide an innovative way to communicate the latest HIV prevention information and support trimestral HIV screening among Latino/a MSM and TGW.

**Trial registration:**

ClinicalTrials.gov, NCT03144336. Registered on 5 May 2017.

## Background

In the United States, men who have sex with men (MSM) and transgender women (TGW) continue to be disproportionally affected by HIV, with racial and ethnic minority MSM and TGW carrying the largest HIV burden. There are few evidence-based effective HIV prevention interventions available for racial and ethnic minority MSM and TGW; biomedical breakthroughs that could offer additional prevention strategies need to be effectively communicated to these populations. Given the quickly changing nature of prevention information and guidelines, a communication framework is required that can be adapted as new information emerges.

Racial and ethnic minority MSM and TGW face unique challenges in accessing and understanding the most up-to-date prevention information. Mobile technologies offer a low-cost yet effective way to remain in contact with these populations; studies suggest that the technologies might be a critical mechanism for relaying HIV-related information [1–5]. Over half (64%) of Americans own a smartphone; Latinos and blacks are three to four times more likely than whites to depend on their smartphone as a primary tool for communication and online access [6]. However, despite the promise of these technologies for enhancing communication with these populations [7–12], studies have noted that participants face multiple challenges when trying to engage with the technologies [13] or abandon them after a short time [14–16].

We are piloting a small randomized controlled trial (RCT) to examine the feasibility and acceptability of integrating mobile technologies and behavioral economics (BE) in a novel way to reach Latino/a MSM and TGW with information about HIV prevention. In the formative phase of this study, focus groups with study participants generated some key lessons for how to use mobile technology with our populations of interest, including: consider requiring ownership of a smartphone (rather than a basic cell phone) to enroll in the study; send messages at the same time and day of the week; allow for personalization of messages; give concrete examples of potential texts so participants know what to expect; and work with a trusted community partner to tailor message structure and content. We also learned that small financial incentives could support participant retention, increase engagement with prevention information and HIV-testing frequency, and support individuals in their goal to remain HIV-negative. We are applying these lessons in designing the current intervention, which we describe here.

BE suggests that small, frequent “nudges” can effectively alter behavior. The “nudge” in our RCT takes the form of a chance to win a prize. Rather than focus on the magnitude of the prize, BE suggests that the way prizes are given out – and at what time intervals – determines their effectiveness. Prize drawings leverage the bias of overestimating small probabilities (leading individuals to participate in the drawing because they overestimate their chance of winning) and also increase salience (frequent prizes keep a behavior high on a person’s mental priority list). Rewards given out through prize drawings also exploit the motivational power and pleasure of games of chance. A number of studies have documented the effectiveness of lotteries in affecting complex health behaviors such as breastfeeding, weight reduction, as well as obesity and cardiovascular disease prevention [17], in addition to targeting sexual behavior [18].

There are few published studies of using prizes for HIV-related behaviors; however, preliminary results from an ongoing study by the principal investigator (PI) [R34MH096609] to improve adherence using small lottery prizes in Uganda show promise. Over the first nine months of that study, individuals receiving incentives were nearly 25% more likely to achieve 90% antiretroviral adherence compared with the control group [19]. Taken together, these studies suggest further exploration of how lotteries support individuals in their effort to maintain their HIV-negative status.

In this pilot RCT, we focus on the local population of Los Angeles County, where Latino/a MSM and TGW are disproportionately impacted by HIV. The estimated HIV prevalence is 40% among Latino MSM [20] and 17% among Latina TGW [21]. MOTIVES—the **Mo**bile **T**echnology and **I**ncenti**ves** intervention—pilots a novel way to reach and support Latino/a MSM and TGW, who are hard to contact and have difficulty accessing relevant information about HIV prevention. We are pairing mobile technologies, a low-cost way to relay the most up-to-date prevention information, with incentives to encourage ongoing participation and translate key prevention messages into healthy behaviors. Our goal is to assess the feasibility and acceptability of the proposed intervention and associated outcome measures among potential participants and program staff to inform a definitive RCT on intervention effectiveness in the future.

## Methods/Design

This pilot study satisfies all checklist items in the CONSORT extension for randomized pilot and feasibility trials by Eldridge and colleagues [22].

### Design

We will pilot test the feasibility and acceptability of the intervention in a small RCT among 200 Latino/a MSM and TGW from Bienestar Human Services, Inc.’s (*Bienestar*) HIV testing sites. There will be approximately 90 MSM and 10 TGW in each arm. Participants will complete a survey at baseline, six, and 12 months. Participants will be randomized (procedures for this are described in detail below) into either the “information only” group (e.g. receiving text messages with HIV prevention information) or the “information plus” group (e.g. additionally receiving quiz questions that provide the possibility of winning prizes when answered correctly). Randomization will provide the comparison needed to identify the potential effect of winning a prize.

We will pilot the recruitment process with five participants. We will begin with a staggered enrollment process based on previous HIV testing data to identify the highest volume *Bienestar* sites. During the study we are collecting quantitative data on both primary outcomes (e.g. HIV knowledge and frequency of testing behavior) and secondary outcomes (e.g. HIV status).

All participating institutions received approval from their respective Institutional Review Boards and a certificate of confidentiality has been obtained from the National Institute of Mental Health. Any protocol modifications will be submitted to the IRBs for review, and participants will be informed if warranted. The trial was registered on 5 May 2017 with the ClinicalTrials.gov registry (NCT03144336).

### Setting

*Bienestar* is a community-based organization offering culturally tailored and linguistically appropriate programs and services to a predominately Latino population. Founded in 1989, *Bienestar* has seven locations in Los Angeles County. The populations served by *Bienestar* include people living with HIV and individuals at risk for HIV, gay and bisexual men, lesbian women, TGW, youth, and injection drug users. Three mobile testing vans provide HIV testing at the agency’s offices and in community venues. The agency provides linkage to HIV medical care for those testing HIV positive. Referrals are also provided to *Bienestar* prevention programs for HIV positive people and to outside agencies for services that *Bienestar* does not provide. *Bienestar* also offers individual-level and group-level interventions focused on reducing the risk of HIV infection or transmission; a syringe exchange program is available for injection drug users. Client services include mental health counseling, housing assistance, nutritional assistance via a food bank, treatment education, and a variety of support groups. *Bienestar* has an extensive history of implementing research, including NIH-funded collaborations with RAND (R01MH072351-05S, R34MH096544-01A1S1, R21DA035629) [21, 22].

### Characteristics of participants

Latino/a MSM and TGW clients who present at *Bienestar* HIV testing sites will undergo risk reduction counseling and rapid HIV testing following usual testing site protocols. Clients who test positive for HIV will be linked to services per the standard of care at *Bienestar*. Clients who test negative for HIV will be asked if they have interest in participating in the MOTIVES study. Those who express interest will be screened for the following eligibility criteria: own or have regular access to a smartphone; self-identify as MSM or TGW; self-identify as a Latino/a; aged ≥ 18 years; fluent in English or Spanish; able to provide contact information for at least three tracking mechanisms (e.g. cell phone, email, address, friend’s contact); and testing HIV negative.

### MOTIVES intervention

**Fig. 1:**
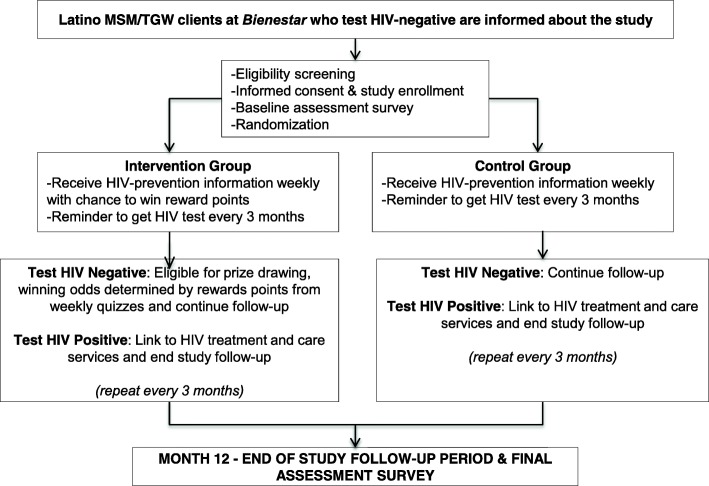
SPIRIT figure

Figure 1 details all stages of the MOTIVES intervention. Participants will be recruited by each tester after they complete their HIV test and the tester assesses that they may be eligible. Other HIV testers who are not part of MOTIVES are aware of the study and will be given flyers with the study information to provide to clients who might be eligible or interested. Eligible clients who express interest will participate in the consent process. Clients will receive a brief overview of the study and a description of the HIV prevention information and prize drawing components. The consent process will also make it clear that only half of the participants will be randomly assigned to the prize drawing group, but that all participants will receive the weekly information messages. Participants will also receive an additional consent document asking them to save the study phone number as “MOTIVES.” If they do not have a lock code on their phone, we will ask if they would like help in creating a code to increase privacy. The study team will confirm that the participants know how to delete messages if they have concerns about someone else reading their messages. The MOTIVES testers will give them information about when to expect text messages from the study (e.g. 2 pm on Thursday every week for one year and a reminder every 2.5 months to test again). The targeted testing frequency is at least once every three months. We will give participants examples of the kinds of text messages and quizzes they will receive and we will explain the odds of winning if they are in the intervention group. The hard copy of the signed consent form will be stored in a locked cabinet at each recruitment site. Following the consent process, the participant will be enrolled in the study by completing the Study Contact form that requires them to provide three tracking mechanisms and assigns them a Study ID number.

At enrollment, we will take several steps to ensure the confidentiality of participants related to text message communication. For example, as mentioned above, MOTIVES HIV testers will instruct participants how to properly use the SMS system, maintain privacy on their device, and delete SMS messages from their mobile device. Finally, study staff will call the participant (either using an office line or using the *67 feature to make a protected call using their personal cell phone) to confirm that study staff has the correct cell phone contact number for the participant. At this time, participants will receive a $15 gift card.

After participating in the baseline survey and receiving their gift card, participants will be randomized using computer allocation by the study PI. The goal is to ensure that the randomization process does not influence responses to the baseline survey (e.g. responding more quickly if they were not chosen for the prize drawing group and feeling disappointed and less invested in the study). The study design was guided by its purpose as a pilot trial; we used a 1:1 randomization as it provides the greatest power for testing effectiveness in preparation for a future definitive RCT. Hereafter we will refer to the two groups as: (1) Information plus group (intervention group); and (2) Information only group (control group). HIV testers will also reaffirm that assignment is completely random and not based on the personal characteristics of any individual client. RAND study staff will explain to testers why randomization is so important, underscoring that only with their help can we actually understand if the intervention works. If testers select participants based on their own judgment of who is most in need of the intervention (e.g. they think a client appears particularly poor and would really appreciate having gift cards), we would not be able to collect accurate data. If a certain type of client is consistently included in the information plus reminders group, we will not be able to determine if the intervention works with everyone or just with a particular type of person.

Informed by findings from the focus groups during the formative phase, we created an incentive system where the chance of winning is high enough, yet not certain, and the prize amount is high enough, yet manageable for our budget. For each 12-week period in which a client is enrolled and has negative HIV test results, the client has a one out of 10 chance of winning a $50 gift card. Over the course of the year, if all four of a client’s HIV tests are negative, the client will have a four out of 10 chance of winning. If the client answered all of the quiz questions correctly, the client will approximately double the chance of winning.

Text messages that address the unique needs of Latino/a MSM and TGW clients will be sent weekly in Spanish or English, according to the participant’s preference. Based on feedback from our focus groups, we will send the initial information on Wednesday and a follow-up question on Friday. The text will include a link to additional information (e.g. CDC website with more information). The weekly information messages will have the following format: “*Hi this is MOTIVES, did you know that [information content]? Use this link [hyperlink] for more information*.” These messages are sent to both study groups. Because we asked participants to save the number from which we will send the messages as “MOTIVES,” they can easily recognize the sender without increasing the chance that study participation reveals their engagement with an HIV prevention exercise.

Messages will be sent once a week; two days later, only participants in the Information plus (i.e. prize drawing) group will receive a message with a question about the information (e.g. “*Is using two condoms safer than using one*?”). The answer option takes the format “*Press 1 if true, press 2 if false to increase your chances of winning a prize.*” This format has been found useful in the PI’s previous text messaging study as it avoids mistakes related to typos and reduces the effort and time needed to respond.

Recent BE studies suggest that giving participants prompt, frequent feedback is critical to changing behavior and can help keep them engaged. Therefore, immediately after participants select an answer by sending “1” or “2” back by text message, they will receive an answer saying either, “*Congratulations, you knew the right answer! You just increased your chances for your next prize drawing*! *Here is a link [hyperlink] to get more information*” or “*That was not the right answer; please follow this [hyperlink] to get more information*.” MOTIVES is designed to promote participant retention and follow-up; therefore, additional incentives will not be provided to engage participants who drop out of the intervention.

### Measures: study acceptability and feasibility

Acceptability (e.g. the extent to which clients receiving the intervention consider it to be appropriate, based on anticipated or experienced cognitive/emotional responses [23]) will be assessed in the following ways. In post-intervention exit interviews (~ 60 min in length), clients will be asked the degree to which they enjoyed participating in the intervention, and why. Clients will also be asked about perceived effectiveness (e.g. How effective do you think the intervention was at improving your knowledge of HIV and HIV testing frequency?) and self-efficacy (e.g. How confident are you that you will be able to continue testing for HIV every 3 months?). Additionally, during the final survey, we will ask participants if and how they benefitted from the intervention and what they could be improved. Finally, intervention will be defined as acceptable if > 50% of potentially eligible participants chose to participate. Feasibility (e.g. the belief that the new behavior is concrete and achievable [24]) will be assessed in the following ways: recruitment rate (number recruited per month compared to number expected to be recruited per month from clinic data), as well as refusal and retention rates (screener, randomization, surveys, and intervention from staff reports). We will assess the feasibility and suitability of eligibility criteria (e.g. whether the criteria are clear and sufficient or too restrictive) by examining the proportion of potential participants who are screened as ineligible and the reasons for ineligibility. We will also consider the feasibility and suitability of the data collection assessments (extent of missing or unusable survey data) and the amount of data collection (time taken to complete surveys from study staff). We will collect sociodemographic characteristics at screening and conduct chi-square or t-tests comparing participants and non-participants. We will supplement refusal and retention quantitative data by asking participants who refuse to participate or who do not complete the intervention about why they chose not to participate in exit interviews. We will transcribe and analyze client exit interviews and reasons for non-participation to further document barriers to acceptability and feasibility.

### Measures: health outcomes

Enrolled participants respond to a baseline assessment after they have completed screening and consent. The MOTIVES HIV tester reads the baseline survey to participants to guarantee understanding by clients across a range of literacy levels. Together the screening and consent processes take approximately 15 min to complete; the baseline survey takes approximately 30 min. Participants will complete a shortened six-month survey via response to a link sent to them on their phone. If participants cannot complete the six-month survey on their own, the Study Coordinator will offer to meet them at a local library of their choice and provide a tablet to complete the survey. However, we expect this circumstance be the exception rather than the rule. Participants will receive a $15 gift certificate via text after completing the six-month survey.

The 12-month follow-up survey will have fewer questions and will be completed after the intervention is over, Thus, completing the questions at a *Bienestar* testing location will not confound the outcome of interest. Participants will receive a $15 gift card for participating in the final survey. For those who do not complete their HIV test for that period, we will send a text message reminder to complete the final survey at *Bienestar*. Table 1 describes when each set of questions will be asked.

**Table 1:** Health outcomes and measures of interest

Outcome	Instrument	Data collection timepoint
Primary Outcome		
HIV knowledge	Carey MP, et al. Development and psychometric evaluation of the brief HIV knowledge questionnaire (hiv-kq-18). AIDS Education and Prevention. 2002; 14: 174-184.	enrollment, 6 & 12 months
HIV testing behaviors	Extracted from *Bienestar* clinic data	
Secondary Outcomes		
HIV infection	Oraquick/Orasure	enrollment, 3, 6, 9 and 12 months
Covariates of Interest		
Socio-demographic information	Based on previous *Bienestar* questionnaire	enrollment & 12 months
Acculturation	Marín, G et al. Development of a short acculturation scale for Hispanics. Hispanic Journal of Behavioral Sciences. 1987; 9:183–205.	
Mental health	Kessler, RC et al. Short screening scales to monitor population prevalences and trends in non-specific psychological distress. Psychological Medicine. 2002; 32: 959-956.	
Substance abuse	Carballo-Diéguez, A et al. Microbicide Safety and Acceptability in Young Men: Baseline Behavioral Questionnaire. 2010.	
Behavioral Economics	Linnemayr et al. Behavioral Economics and Health. 2017

### Data safeguarding and monitoring plan

No drugs will be administered during this study; therefore, the data safeguarding and monitoring plan focuses primarily on confidentiality. We will take the following steps to reduce the risk of disclosure: (1) all study staff will sign a confidentiality agreement requiring them to keep the information obtained in this study private and, if a breach of confidentiality occurs, to report this to the PI within 24 h of discovery; (2) all data will be collected using coded ID numbers; (3) all hard copy materials will be stored in a locked file cabinet/drawer; and (4) only aggregate data that cannot be used to identify individuals will be included in any reports released to other agencies or for publication.

Participants may experience psychological distress or social risks if information that is perceived to be sensitive (e.g. sexual orientation, gender identity, substance abuse, mental health status) is disclosed. Disclosing information about use of illicit drugs may pose legal risks for participants. If a participant exhibits signs of distress, the PI and site coordinator are contacted to ensure that immediate support is provided.

Study staff will be required to report adverse events (AE) or serious adverse events (SAE) to the research team, who will transmit them to the PI immediately. In turn, RAND’s Human Subjects Protection Committee requires the PI to report any AE or SAE within 24 h and will review such events promptly. All the relevant information may not be available at that point. RAND’s Human Subjects Protection Committee will help to determine how serious the event is, what additional information (if any) is needed, and what mitigation procedures should be taken. For example, a misrouting of confidential information from a *Bienestar* staff member to a RAND team member by email instead of a secure file transfer protocol is not a SAE, but quick action to contain the spread of information and delete it correctly can mitigate the potential damage, and some procedural changes and retraining may be needed.

### Data analysis

As previously stated, the primary goal of the analysis is to estimate the intervention’s acceptance and feasibility by determining enrollment and retention rates in the study, using the number of clients screening positive for eligibility, the number of clients enrolling in the study per month, percentage of enrolled clients who respond to the weekly quizzes and are eligible to participate in the testing-based prize drawings, and participants retained in the study. To assess participants’ acceptance of the intervention, we will also ask them to rate their program experience. Semi-structured exit interviews after the study is completed will also provide valuable feedback about the acceptability of the program to clients and the feasibility of the intervention procedures as viewed by clinic personnel.

We will conduct interim data checks to ensure that certain individuals (e.g. either high or low income) are not systematically declining participation in the study. Data checks will be supplemented with bi-weekly team calls in which we review recruitment and retention goals, address issues that arise in the field, and refine our strategy for moving forward. Because most of the study data are uploaded electronically, we avoid issues of double data entry. Interim data checks help ensure the accuracy of data provided.

Blinding participants and staff to the study design raises several issues. Participants will know if they are in the intervention (Information plus group) versus control (Information only group) as the former will be receiving weekly quiz options. However, with the exception of the site PI and the research analyst at RAND responsible for sending all text messages, study staff will be blinded to the data identifiers. All data for subsequent analysis will be de-identified and only the PI and Co-Investigators will have access to the study data. Data will only be unblinded in the case of an AE or SAE, for which the RAND PI needs immediate access to all information to ensure participant safety.

We will conduct group comparisons at six-month intervals to assess the intervention’s short- and mid-term effects. In addition to simple group comparisons at each time point, we will exploit the longitudinal nature of the data by using repeated-measures and time-series techniques. This approach will allow us to examine whether self-reported sexual behaviors persist or decline similarly between the two groups. Repeated measures models will be fitted using Xtreg in the software package Stata. We will use an Analysis of Covariance (ANCOVA) framework to test for group differences, controlling for relevant client characteristics that are found to differentiate the groups at baseline in order to improve the precision that would result from a simple Analysis of Variance (ANOVA) on randomly assigned individuals. For analyses with the dichotomous variables such as HIV testing within a three-months period, we will use a non-parametric McNemar’s test and an analogous multiple logistic regression to control for covariates to assess group differences.

As with any randomized trial (that eliminates selection bias), the first and most direct way to evaluate the impact of an intervention is a simple comparison *D* of the group means in adherence levels.

1. $D=E\left[{Y}_i^T\left|T\right.\right]-E\left[{Y}_i^C\left|C\right.\right]$, *E[Y]* indicates the expected outcome in intervention group *T* and control group *C*.
2. Taking into account the longitudinal nature of the data, this can be written as: $D=E\left[\Delta {Y}_i^T\left|T\right.\right]-E\left[\Delta {Y}_i^C\left|C\right.\right]$
3. In small samples efficiency can be improved by controlling for differences in control variables between the groups that may arise due to chance as discussed above. The approach can be implemented in a regression framework (and again, can be implemented in differences rather than levels): *Y*_*i*_ = *α* + *βT* + *δX*_*i*_ + *ε*_*i*_

As summarized in Table 1, we will explore a range of variables. The independent variables include control variables not only to improve efficiency, but also to learn about the paths through which the intervention works and to identify subgroups that are likely to particularly benefit from the intervention. Furthermore, potential mediators of behavior, such as level of acculturation, may result in improved provider communication and hence information access. For example, some people may want to avoid being seen at a clinic or HIV testing site, but for people who can communicate with providers more easily, this cost is close to zero, and their behavior may not be altered through the intervention. We can test this effect by forming an interaction between the acculturation measure and the intervention. With respect to the dependent variables, the primary outcomes include HIV knowledge and frequency of HIV testing; the secondary outcomes include HIV infection. Continuous variables will be assessed for normality and transformed if needed. Analyses will be conducted for the whole sample as well as for Latino/a MSM and TGW separately to arrive at the potentially differential impact of the MOTIVES intervention for these two groups. Logit estimation will be used for binary outcome measures.

We will impute missing data for some variables if an individual is still enrolled. When participants drop out, we will fit multiple logistic regression models to assess whether this dropout is random. If it is not, we will construct “non-response” weights using logistic regression that correct for dropout. Analyses will reflect these design effects in the calculation of standard errors and tests of significance.

In summary, the primary purposes of our pilot study are to demonstrate the acceptability and feasibility of methods proposed for a subsequent large-scale trial, to stabilize procedures so they are replicable, and to determine important parameters with sufficient accuracy to allow reliable estimates of sample size, power, and detectable effects for the subsequent study. “Mission-critical” parameters include proportions for dichotomous endpoints (such as agreeing to be enrolled in the trial, positive result by HIV rapid test); means and standard deviations for continuous endpoints (such as frequency of repeat HIV-negative test result); and the spectrum of response profiles for longitudinal studies (such as improvement or degradation in retention of HIV prevention information).

## Discussion

There are currently few BE-based interventions in HIV prevention; to our knowledge such interventions have not been used to specifically support the retention of HIV prevention information and increase HIV testing frequency. A key characteristic of our program design is compatibility with any other HIV prevention activities that may be implemented during the course of the study. Thus, rather than being tied to specific content like other interventions, we will create a framework and build a line of interactive communication through which the most up-to-date prevention information will be disseminated and at-risk individuals will be motivated to engage and stay connected with HIV-related care irrespective of the structural barriers they face. Further, given the potential impact of results from this trial, the dissemination strategy includes a diverse set of audiences and multiple dissemination channels such as peer-reviewed journal articles, conferences, as well as community focused events to relay key findings of our research.

Many of the potential barriers to implementing the study (such as issues related to language, cultural appropriateness, smartphone access, or switch in phone numbers during the study period) have been investigated and found to be addressable in a preceding pre-pilot study; we can therefore focus the pilot on finding ways to tailor the information and incentives provided to the specific needs of Latino/a MSM and TGW. To address any remaining challenges, the study team will meet regularly to find acceptable solutions, which we will also discuss with *Bienestar* and its Community Advisory Committee.

If we demonstrate that this BE-informed, mobile technology-based intervention is acceptable, feasible, and cost-effective, it will establish a new way of providing incentives to improve HIV prevention outcomes. The study will provide a formative assessment of the intervention’s acceptability and feasibility and will also inform ways to optimize and tailor the intervention for a future RCT of intervention effectiveness. Findings may have implications not only for Latino/a MSM and TGW in Los Angeles, California, but also for other racial and ethnic minority groups disproportionately impacted by HIV.

## Trial status

**Protocol version:** Final version as of 12 November 2017.

**Recruitment dates:** The date of first enrollment was 31 May 2017 and participants are currently being recruited and enrolled through 9 May 2018.

**Enrollment status:** Active.

## Abbreviations

AE: Adverse events
ANCOVA: Analysis of covariance
ANOVA: Analysis of variance
BE: Behavioral economics
CBO: Community-based organization
DSMP: Data safeguarding and monitoring plan
HIV: Human immunodeficiency virus
ID: Identification
IRB: Institutional review board
MOTIVES: Mobile technology and incentives
MSM: Men who have sex with men
NIH: National Institutes of Health
PI: Principal investigator
RCT: Randomized control trial
SAE: Serious adverse events
SMS: Short messaging system
TGW: Transgender women
